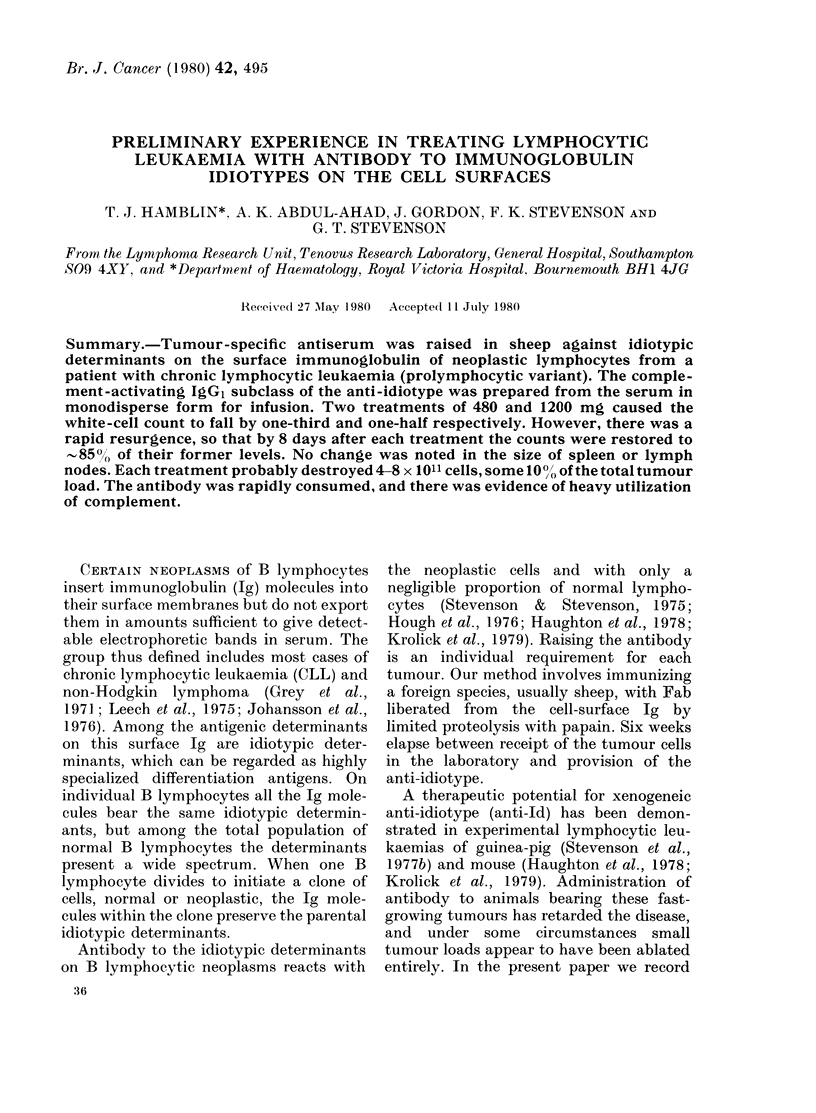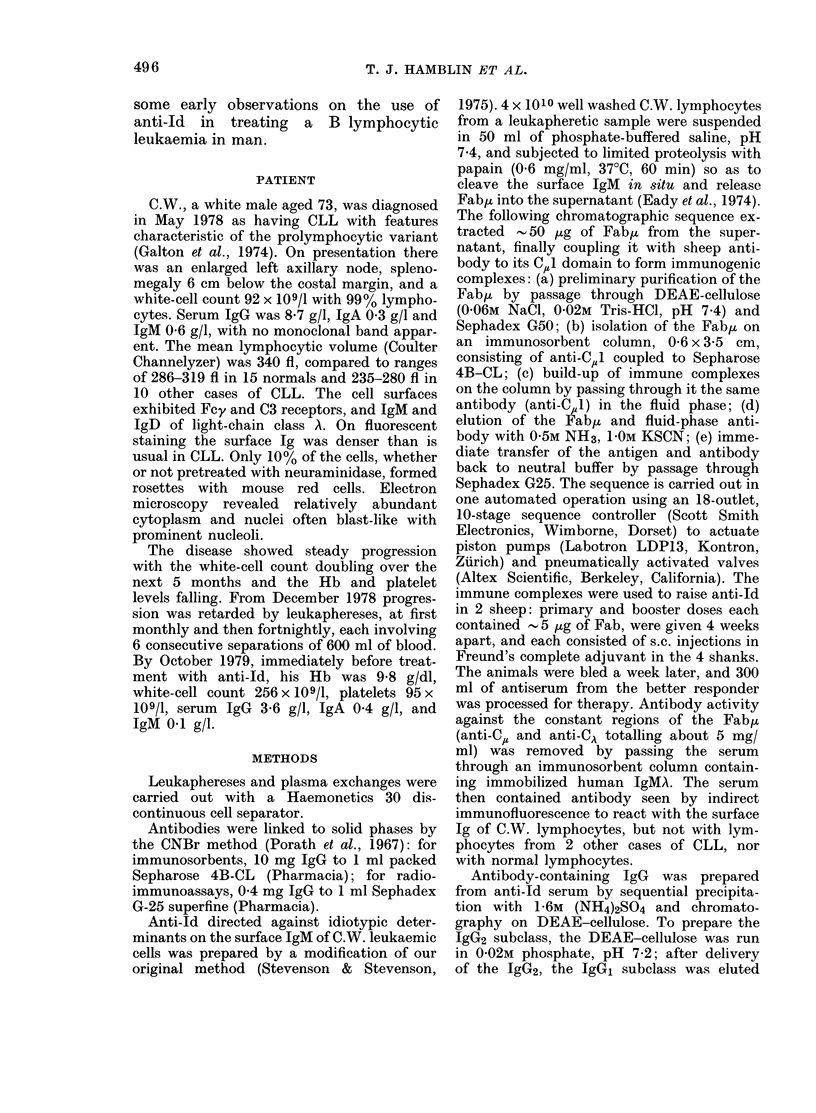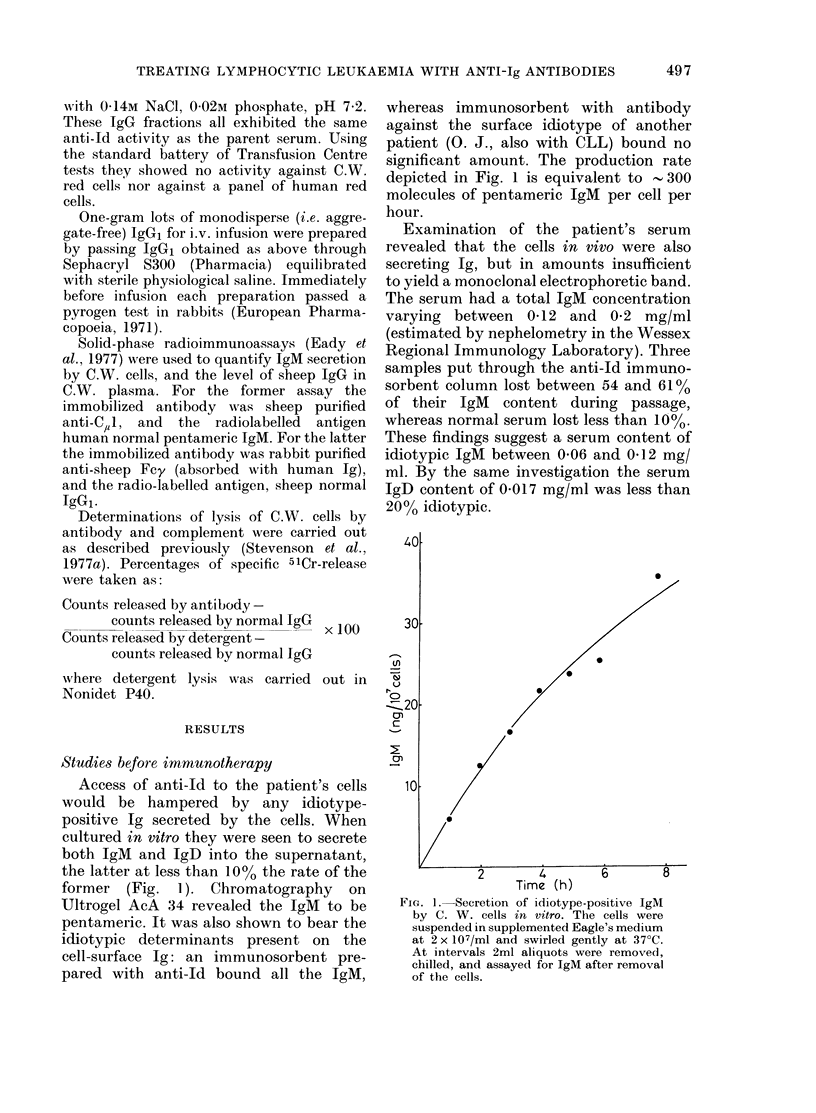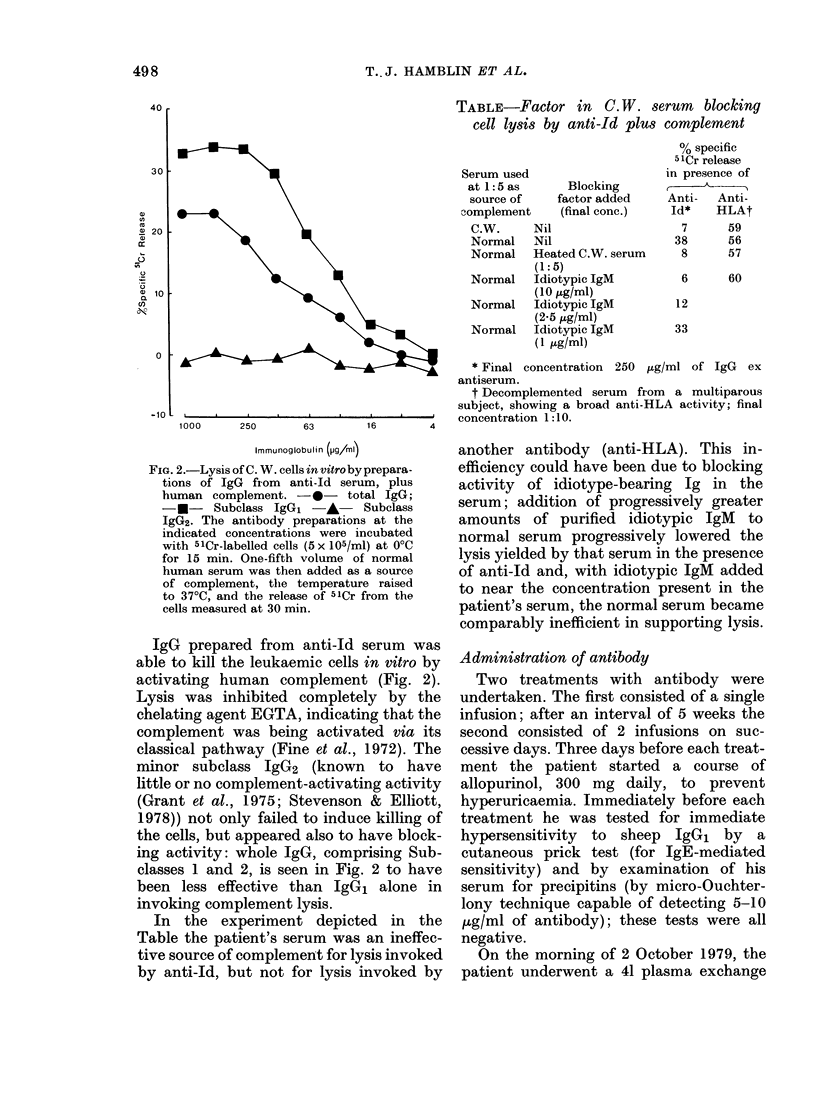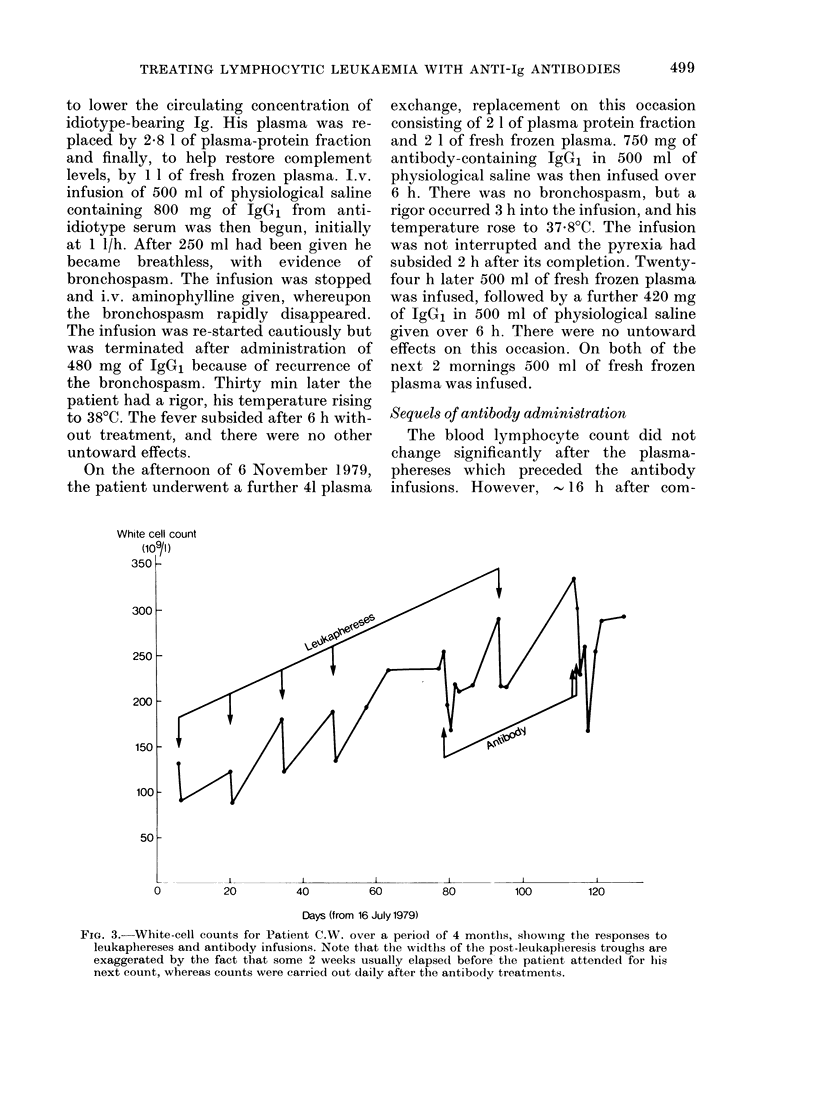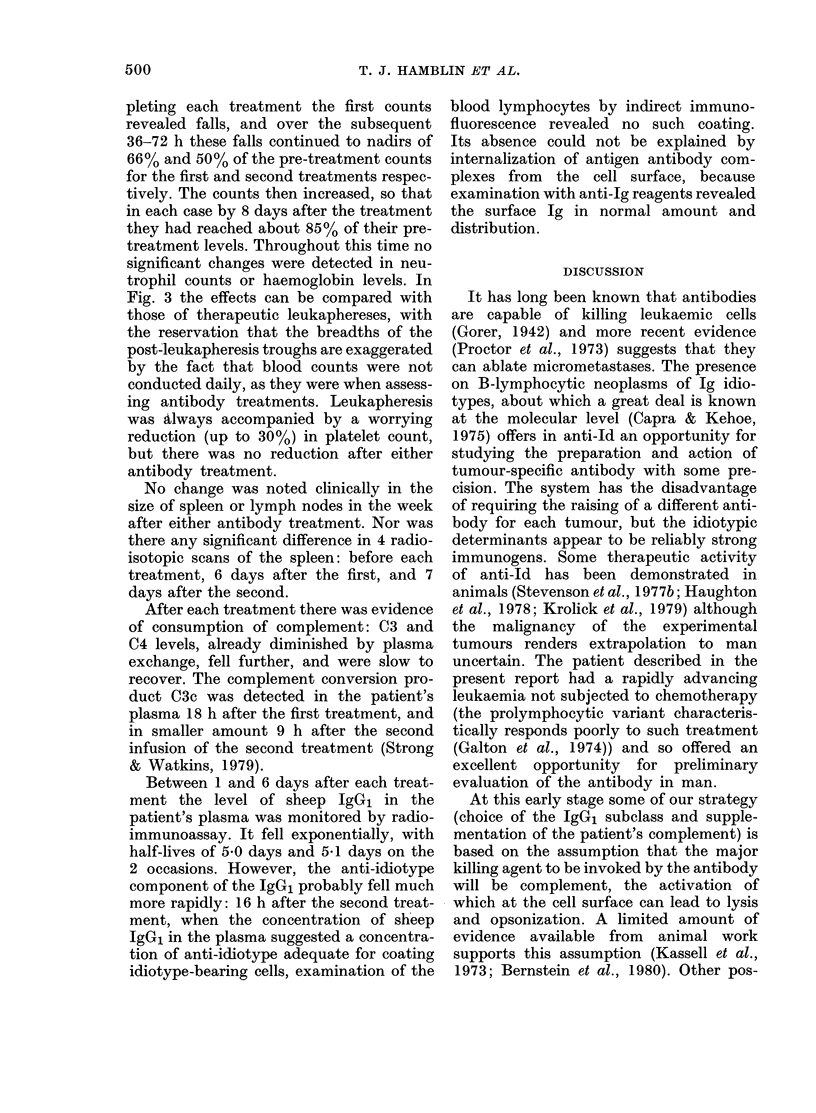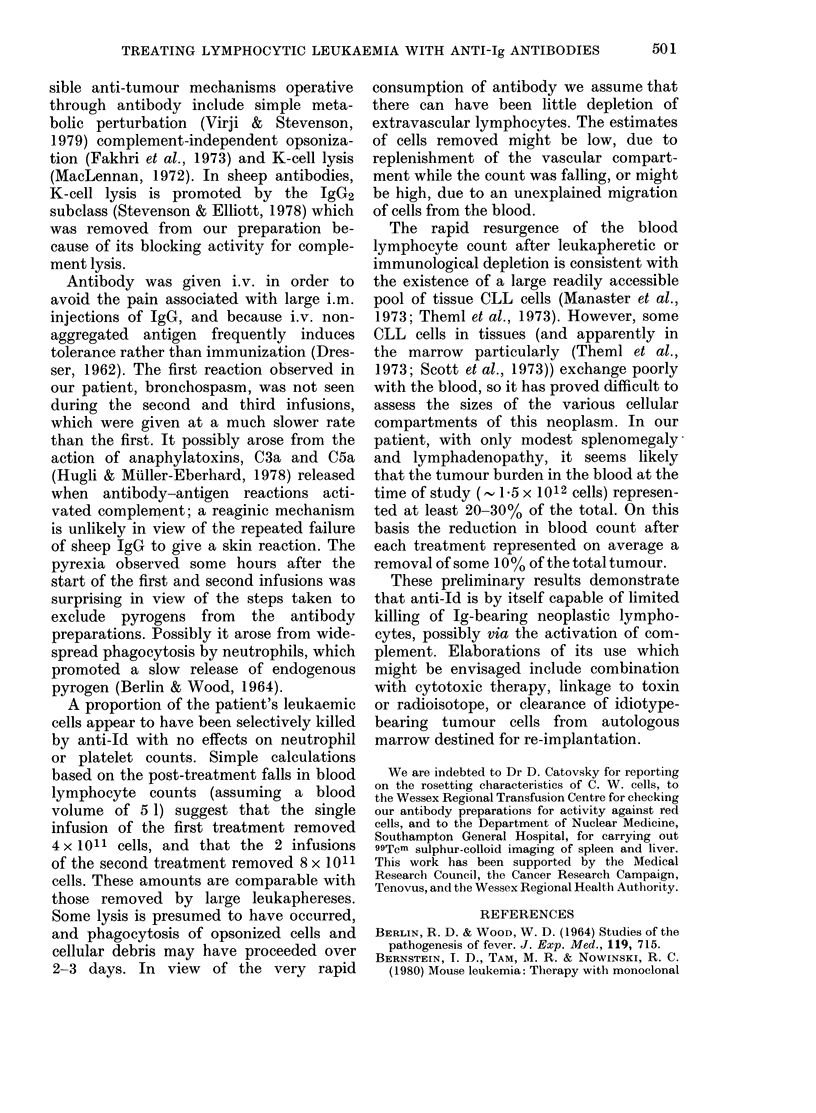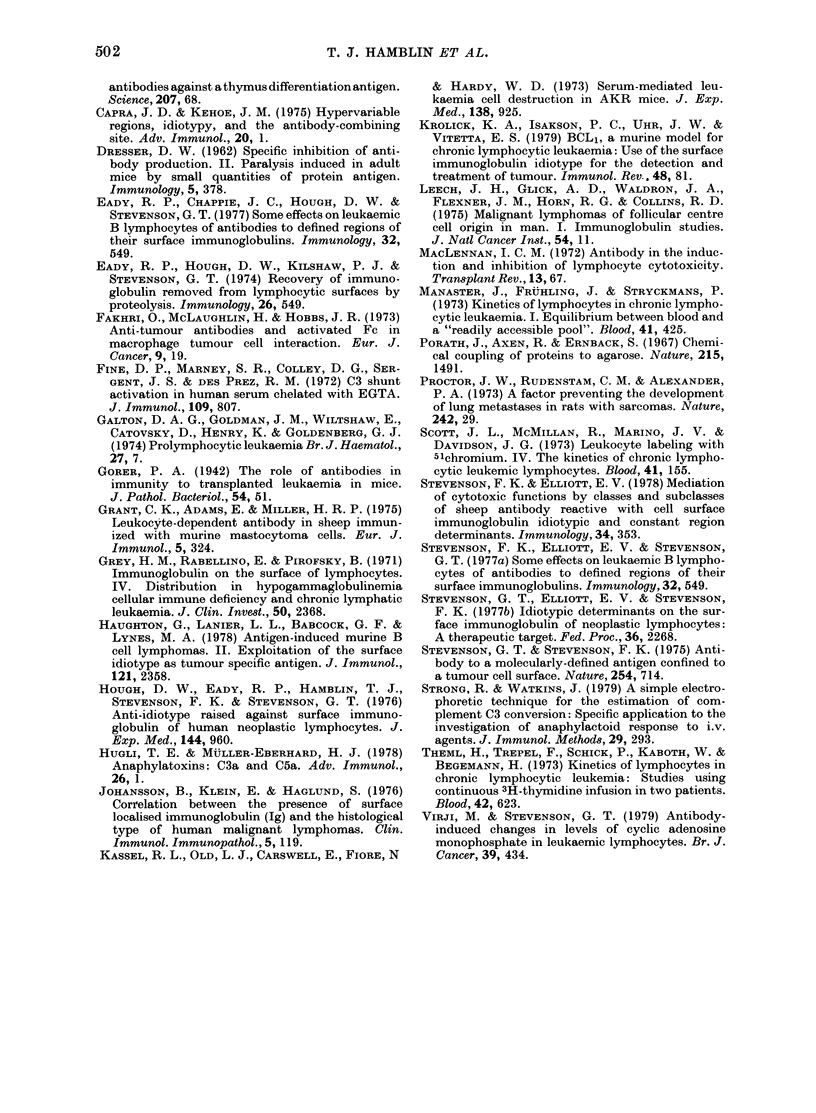# Preliminary experience in treating lymphocytic leukaemia with antibody to immunoglobulin idiotypes on the cell surfaces.

**DOI:** 10.1038/bjc.1980.271

**Published:** 1980-10

**Authors:** T. J. Hamblin, A. K. Abdul-Ahad, J. Gordon, F. K. Stevenson, G. T. Stevenson

## Abstract

Tumour-specific antiserum was raised in sheep against idiotypic determinants on the surface immunoglobulin of neoplastic lymphocytes from a patient with chronic lymphocytic leukaemia (prolymphocytic variant). The complement-activating IgG1 subclass of the anti-idiotype was prepared from the serum in monodisperse form for infusion. Two treatments of 480 and 1200 mg caused the white-cell count to fall by one-third and one-half respectively. However, there was a rapid resurgence, so that by 8 days after each treatment the counts were restored to approximately 85% of their former levels. No change was noted in the size of spleen or lymph nodes. Each treatment probably destroyed 4-8 X 10(11) cells, some 10% of the total tumour load. The antibody was rapidly consumed, and there was evidence of heavy utilization of complement.


*Br. J. Cancer* (1980) **42**, 495

### PRELIMINARY EXPERIENCE IN TREATING LYMPHOCYTIC LEUKAEMIA WITH ANTIBODY TO IMMUNOGLOBULIN IDIOTYPES ON THE CELL SURFACES

T. J. HAMBLIN*, A. K. ABDUL-AHAD, J. GORDON, F. K. STEVENSON AND G. T. STEVENSON

*From the Lymphoma Research Unit, Tenovus Research Laboratory, General Hospital, Southampton SO9 4XY, and *Department of Haematology, Royal Victoria Hospital, Bournemouth BH1 4JG*

Received 27 May 1980 Accepted 11 July 1980

**Summary.—Tumour-specific antiserum was raised in sheep against idiotypic determinants on the surface immunoglobulin of neoplastic lymphocytes from a patient with chronic lymphocytic leukaemia (prolymphocytic variant). The complement-activating $IgG_1$ subclass of the anti-idiotype was prepared from the serum in monodisperse form for infusion. Two treatments of 480 and 1200 mg caused the white-cell count to fall by one-third and one-half respectively. However, there was a rapid resurgence, so that by 8 days after each treatment the counts were restored to ~85% of their former levels. No change was noted in the size of spleen or lymph nodes. Each treatment probably destroyed $4–8 \times 10^{11}$ cells, some 10% of the total tumour load. The antibody was rapidly consumed, and there was evidence of heavy utilization of complement.**

CERTAIN NEOPLASMS of B lymphocytes insert immunoglobulin (Ig) molecules into their surface membranes but do not export them in amounts sufficient to give detectable electrophoretic bands in serum. The group thus defined includes most cases of chronic lymphocytic leukaemia (CLL) and non-Hodgkin lymphoma (Grey *et al.*, 1971; Leech *et al.*, 1975; Johansson *et al.*, 1976). Among the antigenic determinants on this surface Ig are idiotypic determinants, which can be regarded as highly specialized differentiation antigens. On individual B lymphocytes all the Ig molecules bear the same idiotypic determinants, but among the total population of normal B lymphocytes the determinants present a wide spectrum. When one B lymphocyte divides to initiate a clone of cells, normal or neoplastic, the Ig molecules within the clone preserve the parental idiotypic determinants.

Antibody to the idiotypic determinants on B lymphocytic neoplasms reacts with the neoplastic cells and with only a negligible proportion of normal lymphocytes (Stevenson & Stevenson, 1975; Hough *et al.*, 1976; Haughton *et al.*, 1978; Krolick *et al.*, 1979). Raising the antibody is an individual requirement for each tumour. Our method involves immunizing a foreign species, usually sheep, with Fab liberated from the cell-surface Ig by limited proteolysis with papain. Six weeks elapse between receipt of the tumour cells in the laboratory and provision of the anti-idiotype.

A therapeutic potential for xenogeneic anti-idiotype (anti-Id) has been demonstrated in experimental lymphocytic leukaemias of guinea-pig (Stevenson *et al.*, 1977*b*) and mouse (Haughton *et al.*, 1978; Krolick *et al.*, 1979). Administration of antibody to animals bearing these fast-growing tumours has retarded the disease, and under some circumstances small tumour loads appear to have been ablated entirely. In the present paper we record some early observations on the use of anti-Id in treating a B lymphocytic leukaemia in man.

### PATIENT

C.W., a white male aged 73, was diagnosed in May 1978 as having CLL with features characteristic of the prolymphocytic variant (Galton *et al.*, 1974). On presentation there was an enlarged left axillary node, splenomegaly 6 cm below the costal margin, and a white-cell count $92 \times 10^9$/l with 99% lymphocytes. Serum IgG was 8·7 g/l, IgA 0·3 g/l and IgM 0·6 g/l, with no monoclonal band apparent. The mean lymphocytic volume (Coulter Channelyzer) was 340 fl, compared to ranges of 286–319 fl in 15 normals and 235–280 fl in 10 other cases of CLL. The cell surfaces exhibited $Fc\gamma$ and C3 receptors, and IgM and IgD of light-chain class $\lambda$. On fluorescent staining the surface Ig was denser than is usual in CLL. Only 10% of the cells, whether or not pretreated with neuraminidase, formed rosettes with mouse red cells. Electron microscopy revealed relatively abundant cytoplasm and nuclei often blast-like with prominent nucleoli.

The disease showed steady progression with the white-cell count doubling over the next 5 months and the Hb and platelet levels falling. From December 1978 progression was retarded by leukaphereses, at first monthly and then fortnightly, each involving 6 consecutive separations of 600 ml of blood. By October 1979, immediately before treatment with anti-Id, his Hb was 9·8 g/dl, white-cell count $256 \times 10^9$/l, platelets $95 \times 10^9$/l, serum IgG 3·6 g/l, IgA 0·4 g/l, and IgM 0·1 g/l.

### METHODS

Leukaphereses and plasma exchanges were carried out with a Haemonetics 30 discontinuous cell separator.

Antibodies were linked to solid phases by the CNBr method (Porath *et al.*, 1967): for immunosorbents, 10 mg IgG to 1 ml packed Sepharose 4B-CL (Pharmacia); for radioimmunoassays, 0·4 mg IgG to 1 ml Sephadex G-25 superfine (Pharmacia).

Anti-Id directed against idiotypic determinants on the surface IgM of C.W. leukaemic cells was prepared by a modification of our original method (Stevenson & Stevenson, 1975). $4 \times 10^{10}$ well washed C.W. lymphocytes from a leukapheretic sample were suspended in 50 ml of phosphate-buffered saline, pH 7·4, and subjected to limited proteolysis with papain (0·6 mg/ml, 37°C, 60 min) so as to cleave the surface IgM *in situ* and release $Fab\mu$ into the supernatant (Eady *et al.*, 1974). The following chromatographic sequence extracted $\sim$50 $\mu$g of $Fab\mu$ from the supernatant, finally coupling it with sheep antibody to its $C_\mu 1$ domain to form immunogenic complexes: (a) preliminary purification of the $Fab\mu$ by passage through DEAE-cellulose (0·06M NaCl, 0·02M Tris-HCl, pH 7·4) and Sephadex G50; (b) isolation of the $Fab\mu$ on an immunosorbent column, $0{\cdot}6 \times 3{\cdot}5$ cm, consisting of anti-$C_\mu 1$ coupled to Sepharose 4B–CL; (c) build-up of immune complexes on the column by passing through it the same antibody (anti-$C_\mu 1$) in the fluid phase; (d) elution of the $Fab\mu$ and fluid-phase antibody with 0·5M $NH_3$, 1·0M KSCN; (e) immediate transfer of the antigen and antibody back to neutral buffer by passage through Sephadex G25. The sequence is carried out in one automated operation using an 18-outlet, 10-stage sequence controller (Scott Smith Electronics, Wimborne, Dorset) to actuate piston pumps (Labotron LDP13, Kontron, Zürich) and pneumatically activated valves (Altex Scientific, Berkeley, California). The immune complexes were used to raise anti-Id in 2 sheep: primary and booster doses each contained $\sim$5 $\mu$g of Fab, were given 4 weeks apart, and each consisted of s.c. injections in Freund's complete adjuvant in the 4 shanks. The animals were bled a week later, and 300 ml of antiserum from the better responder was processed for therapy. Antibody activity against the constant regions of the $Fab\mu$ (anti-$C_\mu$ and anti-$C_\lambda$ totalling about 5 mg/ml) was removed by passing the serum through an immunosorbent column containing immobilized human $IgM\lambda$. The serum then contained antibody seen by indirect immunofluorescence to react with the surface Ig of C.W. lymphocytes, but not with lymphocytes from 2 other cases of CLL, nor with normal lymphocytes.

Antibody-containing IgG was prepared from anti-Id serum by sequential precipitation with 1·6M $(NH_4)_2SO_4$ and chromatography on DEAE–cellulose. To prepare the $IgG_2$ subclass, the DEAE–cellulose was run in 0·02M phosphate, pH 7·2; after delivery of the $IgG_2$, the $IgG_1$ subclass was eluted with 0·14M NaCl, 0·02M phosphate, pH 7·2. These IgG fractions all exhibited the same anti-Id activity as the parent serum. Using the standard battery of Transfusion Centre tests they showed no activity against C.W. red cells nor against a panel of human red cells.

One-gram lots of monodisperse (*i.e.* aggregate-free) $IgG_1$ for i.v. infusion were prepared by passing $IgG_1$ obtained as above through Sephacryl S300 (Pharmacia) equilibrated with sterile physiological saline. Immediately before infusion each preparation passed a pyrogen test in rabbits (European Pharmacopoeia, 1971).

Solid-phase radioimmunoassays (Eady *et al.*, 1977) were used to quantify IgM secretion by C.W. cells, and the level of sheep IgG in C.W. plasma. For the former assay the immobilized antibody was sheep purified anti-$C_{\mu}1$, and the radiolabelled antigen human normal pentameric IgM. For the latter the immobilized antibody was rabbit purified anti-sheep Fc$\gamma$ (absorbed with human Ig), and the radio-labelled antigen, sheep normal $IgG_1$.

Determinations of lysis of C.W. cells by antibody and complement were carried out as described previously (Stevenson *et al.*, 1977*a*). Percentages of specific $^{51}$Cr-release were taken as:

$$\frac{\text{Counts released by antibody} - \text{counts released by normal IgG}}{\text{Counts released by detergent} - \text{counts released by normal IgG}} \times 100$$

where detergent lysis was carried out in Nonidet P40.

### RESULTS

### *Studies before immunotherapy*

Access of anti-Id to the patient's cells would be hampered by any idiotype-positive Ig secreted by the cells. When cultured *in vitro* they were seen to secrete both IgM and IgD into the supernatant, the latter at less than 10% the rate of the former (Fig. 1). Chromatography on Ultrogel AcA 34 revealed the IgM to be pentameric. It was also shown to bear the idiotypic determinants present on the cell-surface Ig: an immunosorbent prepared with anti-Id bound all the IgM, whereas immunosorbent with antibody against the surface idiotype of another patient (O. J., also with CLL) bound no significant amount. The production rate depicted in Fig. 1 is equivalent to $\sim 300$ molecules of pentameric IgM per cell per hour.

Examination of the patient's serum revealed that the cells *in vivo* were also secreting Ig, but in amounts insufficient to yield a monoclonal electrophoretic band. The serum had a total IgM concentration varying between 0·12 and 0·2 mg/ml (estimated by nephelometry in the Wessex Regional Immunology Laboratory). Three samples put through the anti-Id immunosorbent column lost between 54 and 61% of their IgM content during passage, whereas normal serum lost less than 10%. These findings suggest a serum content of idiotypic IgM between 0·06 and 0·12 mg/ml. By the same investigation the serum IgD content of 0·017 mg/ml was less than 20% idiotypic.

FIG. 1.—Secretion of idiotype-positive IgM by C. W. cells *in vitro*. The cells were suspended in supplemented Eagle's medium at $2 \times 10^7$/ml and swirled gently at 37°C. At intervals 2ml aliquots were removed, chilled, and assayed for IgM after removal of the cells.

FIG. 2.—Lysis of C. W. cells *in vitro* by preparations of IgG from anti-Id serum, plus human complement. —●— total IgG; —■— Subclass $IgG_1$ —▲— Subclass $IgG_2$. The antibody preparations at the indicated concentrations were incubated with $^{51}$Cr-labelled cells ($5 \times 10^5$/ml) at 0°C for 15 min. One-fifth volume of normal human serum was then added as a source of complement, the temperature raised to 37°C, and the release of $^{51}$Cr from the cells measured at 30 min.

IgG prepared from anti-Id serum was able to kill the leukaemic cells *in vitro* by activating human complement (Fig. 2). Lysis was inhibited completely by the chelating agent EGTA, indicating that the complement was being activated *via* its classical pathway (Fine *et al.*, 1972). The minor subclass $IgG_2$ (known to have little or no complement-activating activity (Grant *et al.*, 1975; Stevenson & Elliott, 1978)) not only failed to induce killing of the cells, but appeared also to have blocking activity: whole IgG, comprising Subclasses 1 and 2, is seen in Fig. 2 to have been less effective than $IgG_1$ alone in invoking complement lysis.

In the experiment depicted in the Table the patient's serum was an ineffective source of complement for lysis invoked by anti-Id, but not for lysis invoked by

TABLE—*Factor in C.W. serum blocking cell lysis by anti-Id plus complement*

| Serum used at 1:5 as source of complement | Blocking factor added (final conc.) | % specific $^{51}$Cr release in presence of Anti-Id* | Anti-HLA† |
|---|---|---|---|
| C.W. | Nil | 7 | 59 |
| Normal | Nil | 38 | 56 |
| Normal | Heated C.W. serum (1:5) | 8 | 57 |
| Normal | Idiotypic IgM (10 μg/ml) | 6 | 60 |
| Normal | Idiotypic IgM (2·5 μg/ml) | 12 | |
| Normal | Idiotypic IgM (1 μg/ml) | 33 | |

* Final concentration 250 μg/ml of IgG ex antiserum.

† Decomplemented serum from a multiparous subject, showing a broad anti-HLA activity; final concentration 1:10.

another antibody (anti-HLA). This inefficiency could have been due to blocking activity of idiotype-bearing Ig in the serum; addition of progressively greater amounts of purified idiotypic IgM to normal serum progressively lowered the lysis yielded by that serum in the presence of anti-Id and, with idiotypic IgM added to near the concentration present in the patient's serum, the normal serum became comparably inefficient in supporting lysis.

*Administration of antibody*

Two treatments with antibody were undertaken. The first consisted of a single infusion; after an interval of 5 weeks the second consisted of 2 infusions on successive days. Three days before each treatment the patient started a course of allopurinol, 300 mg daily, to prevent hyperuricaemia. Immediately before each treatment he was tested for immediate hypersensitivity to sheep $IgG_1$ by a cutaneous prick test (for IgE-mediated sensitivity) and by examination of his serum for precipitins (by micro-Ouchterlony technique capable of detecting 5–10 μg/ml of antibody); these tests were all negative.

On the morning of 2 October 1979, the patient underwent a 4l plasma exchange to lower the circulating concentration of idiotype-bearing Ig. His plasma was replaced by 2·8 l of plasma-protein fraction and finally, to help restore complement levels, by 1 l of fresh frozen plasma. I.v. infusion of 500 ml of physiological saline containing 800 mg of $IgG_1$ from anti-idiotype serum was then begun, initially at 1 l/h. After 250 ml had been given he became breathless, with evidence of bronchospasm. The infusion was stopped and i.v. aminophylline given, whereupon the bronchospasm rapidly disappeared. The infusion was re-started cautiously but was terminated after administration of 480 mg of $IgG_1$ because of recurrence of the bronchospasm. Thirty min later the patient had a rigor, his temperature rising to 38°C. The fever subsided after 6 h without treatment, and there were no other untoward effects.

On the afternoon of 6 November 1979, the patient underwent a further 4l plasma exchange, replacement on this occasion consisting of 2 l of plasma protein fraction and 2 l of fresh frozen plasma. 750 mg of antibody-containing $IgG_1$ in 500 ml of physiological saline was then infused over 6 h. There was no bronchospasm, but a rigor occurred 3 h into the infusion, and his temperature rose to 37·8°C. The infusion was not interrupted and the pyrexia had subsided 2 h after its completion. Twenty-four h later 500 ml of fresh frozen plasma was infused, followed by a further 420 mg of $IgG_1$ in 500 ml of physiological saline given over 6 h. There were no untoward effects on this occasion. On both of tbe next 2 mornings 500 ml of fresh frozen plasma was infused.

*Sequels of antibody administration*

The blood lymphocyte count did not change significantly after the plasmaphereses which preceded the antibody infusions. However, ~16 h after com-

FIG. 3.—White-cell counts for Patient C.W. over a period of 4 months, showing the responses to leukaphereses and antibody infusions. Note that the widths of the post-leukapheresis troughs are exaggerated by the fact that some 2 weeks usually elapsed before the patient attended for his next count, whereas counts were carried out daily after the antibody treatments.

pleting each treatment the first counts revealed falls, and over the subsequent 36–72 h these falls continued to nadirs of 66% and 50% of the pre-treatment counts for the first and second treatments respectively. The counts then increased, so that in each case by 8 days after the treatment they had reached about 85% of their pre-treatment levels. Throughout this time no significant changes were detected in neutrophil counts or haemoglobin levels. In Fig. 3 the effects can be compared with those of therapeutic leukaphereses, with the reservation that the breadths of the post-leukapheresis troughs are exaggerated by the fact that blood counts were not conducted daily, as they were when assessing antibody treatments. Leukapheresis was always accompanied by a worrying reduction (up to 30%) in platelet count, but there was no reduction after either antibody treatment.

No change was noted clinically in the size of spleen or lymph nodes in the week after either antibody treatment. Nor was there any significant difference in 4 radioisotopic scans of the spleen: before each treatment, 6 days after the first, and 7 days after the second.

After each treatment there was evidence of consumption of complement: C3 and C4 levels, already diminished by plasma exchange, fell further, and were slow to recover. The complement conversion product C3c was detected in the patient's plasma 18 h after the first treatment, and in smaller amount 9 h after the second infusion of the second treatment (Strong & Watkins, 1979).

Between 1 and 6 days after each treatment the level of sheep $IgG_1$ in the patient's plasma was monitored by radioimmunoassay. It fell exponentially, with half-lives of 5·0 days and 5·1 days on the 2 occasions. However, the anti-idiotype component of the $IgG_1$ probably fell much more rapidly: 16 h after the second treatment, when the concentration of sheep $IgG_1$ in the plasma suggested a concentration of anti-idiotype adequate for coating idiotype-bearing cells, examination of the blood lymphocytes by indirect immunofluorescence revealed no such coating. Its absence could not be explained by internalization of antigen antibody complexes from the cell surface, because examination with anti-Ig reagents revealed the surface Ig in normal amount and distribution.

### DISCUSSION

It has long been known that antibodies are capable of killing leukaemic cells (Gorer, 1942) and more recent evidence (Proctor *et al.*, 1973) suggests that they can ablate micrometastases. The presence on B-lymphocytic neoplasms of Ig idiotypes, about which a great deal is known at the molecular level (Capra & Kehoe, 1975) offers in anti-Id an opportunity for studying the preparation and action of tumour-specific antibody with some precision. The system has the disadvantage of requiring the raising of a different antibody for each tumour, but the idiotypic determinants appear to be reliably strong immunogens. Some therapeutic activity of anti-Id has been demonstrated in animals (Stevenson *et al.*, 1977*b*; Haughton *et al.*, 1978; Krolick *et al.*, 1979) although the malignancy of the experimental tumours renders extrapolation to man uncertain. The patient described in the present report had a rapidly advancing leukaemia not subjected to chemotherapy (the prolymphocytic variant characteristically responds poorly to such treatment (Galton *et al.*, 1974)) and so offered an excellent opportunity for preliminary evaluation of the antibody in man.

At this early stage some of our strategy (choice of the $IgG_1$ subclass and supplementation of the patient's complement) is based on the assumption that the major killing agent to be invoked by the antibody will be complement, the activation of which at the cell surface can lead to lysis and opsonization. A limited amount of evidence available from animal work supports this assumption (Kassell *et al.*, 1973; Bernstein *et al.*, 1980). Other possible anti-tumour mechanisms operative through antibody include simple metabolic perturbation (Virji & Stevenson, 1979) complement-independent opsonization (Fakhri *et al.*, 1973) and K-cell lysis (MacLennan, 1972). In sheep antibodies, K-cell lysis is promoted by the $IgG_2$ subclass (Stevenson & Elliott, 1978) which was removed from our preparation because of its blocking activity for complement lysis.

Antibody was given i.v. in order to avoid the pain associated with large i.m. injections of IgG, and because i.v. non-aggregated antigen frequently induces tolerance rather than immunization (Dresser, 1962). The first reaction observed in our patient, bronchospasm, was not seen during the second and third infusions, which were given at a much slower rate than the first. It possibly arose from the action of anaphylatoxins, C3a and C5a (Hugli & Müller-Eberhard, 1978) released when antibody–antigen reactions activated complement; a reaginic mechanism is unlikely in view of the repeated failure of sheep IgG to give a skin reaction. The pyrexia observed some hours after the start of the first and second infusions was surprising in view of the steps taken to exclude pyrogens from the antibody preparations. Possibly it arose from widespread phagocytosis by neutrophils, which promoted a slow release of endogenous pyrogen (Berlin & Wood, 1964).

A proportion of the patient's leukaemic cells appear to have been selectively killed by anti-Id with no effects on neutrophil or platelet counts. Simple calculations based on the post-treatment falls in blood lymphocyte counts (assuming a blood volume of 5 l) suggest that the single infusion of the first treatment removed $4 \times 10^{11}$ cells, and that the 2 infusions of the second treatment removed $8 \times 10^{11}$ cells. These amounts are comparable with those removed by large leukaphereses. Some lysis is presumed to have occurred, and phagocytosis of opsonized cells and cellular debris may have proceeded over 2–3 days. In view of the very rapid consumption of antibody we assume that there can have been little depletion of extravascular lymphocytes. The estimates of cells removed might be low, due to replenishment of the vascular compartment while the count was falling, or might be high, due to an unexplained migration of cells from the blood.

The rapid resurgence of the blood lymphocyte count after leukapheretic or immunological depletion is consistent with the existence of a large readily accessible pool of tissue CLL cells (Manaster *et al.*, 1973; Theml *et al.*, 1973). However, some CLL cells in tissues (and apparently in the marrow particularly (Theml *et al.*, 1973; Scott *et al.*, 1973)) exchange poorly with the blood, so it has proved difficult to assess the sizes of the various cellular compartments of this neoplasm. In our patient, with only modest splenomegaly and lymphadenopathy, it seems likely that the tumour burden in the blood at the time of study ($\sim 1{\cdot}5 \times 10^{12}$ cells) represented at least 20–30% of the total. On this basis the reduction in blood count after each treatment represented on average a removal of some 10% of the total tumour.

These preliminary results demonstrate that anti-Id is by itself capable of limited killing of Ig-bearing neoplastic lymphocytes, possibly *via* the activation of complement. Elaborations of its use which might be envisaged include combination with cytotoxic therapy, linkage to toxin or radioisotope, or clearance of idiotype-bearing tumour cells from autologous marrow destined for re-implantation.

We are indebted to Dr D. Catovsky for reporting on the rosetting characteristics of C. W. cells, to the Wessex Regional Transfusion Centre for checking our antibody preparations for activity against red cells, and to the Department of Nuclear Medicine, Southampton General Hospital, for carrying out $^{99}Tc^{m}$ sulphur-colloid imaging of spleen and liver. This work has been supported by the Medical Research Council, the Cancer Research Campaign, Tenovus, and the Wessex Regional Health Authority.

### REFERENCES

BERLIN, R. D. & WOOD, W. D. (1964) Studies of the pathogenesis of fever. *J. Exp. Med.*, **119**, 715.

BERNSTEIN, I. D., TAM, M. R. & NOWINSKI, R. C. (1980) Mouse leukemia: Therapy with monoclonal antibodies against a thymus differentiation antigen. *Science*, **207**, 68.

CAPRA, J. D. & KEHOE, J. M. (1975) Hypervariable regions, idiotypy, and the antibody-combining site. *Adv. Immunol.*, **20**, 1.

DRESSER, D. W. (1962) Specific inhibition of antibody production. II. Paralysis induced in adult mice by small quantities of protein antigen. *Immunology*, **5**, 378.

EADY, R. P., CHAPPIE, J. C., HOUGH, D. W. & STEVENSON, G. T. (1977) Some effects on leukaemic B lymphocytes of antibodies to defined regions of their surface immunoglobulins. *Immunology*, **32**, 549.

EADY, R. P., HOUGH, D. W., KILSHAW, P. J. & STEVENSON, G. T. (1974) Recovery of immunoglobulin removed from lymphocytic surfaces by proteolysis. *Immunology*, **26**, 549.

FAKHRI, O., MCLAUGHLIN, H. & HOBBS, J. R. (1973) Anti-tumour antibodies and activated Fc in macrophage tumour cell interaction. *Eur. J. Cancer*, **9**, 19.

FINE, D. P., MARNEY, S. R., COLLEY, D. G., SERGENT, J. S. & DES PREZ, R. M. (1972) C3 shunt activation in human serum chelated with EGTA. *J. Immunol.*, **109**, 807.

GALTON, D. A. G., GOLDMAN, J. M., WILTSHAW, E., CATOVSKY, D., HENRY, K. & GOLDENBERG, G. J. (1974) Prolymphocytic leukaemia *Br. J. Haematol.*, **27**, 7.

GORER, P. A. (1942) The role of antibodies in immunity to transplanted leukaemia in mice. *J. Pathol. Bacteriol.*, **54**, 51.

GRANT, C. K., ADAMS, E. & MILLER, H. R. P. (1975) Leukocyte-dependent antibody in sheep immunized with murine mastocytoma cells. *Eur. J. Immunol.*, **5**, 324.

GREY, H. M., RABELLINO, E. & PIROFSKY, B. (1971) Immunoglobulin on the surface of lymphocytes. IV. Distribution in hypogammaglobulinemia cellular immune deficiency and chronic lymphatic leukaemia. *J. Clin. Invest.*, **50**, 2368.

HAUGHTON, G., LANIER, L. L., BABCOCK, G. F. & LYNES, M. A. (1978) Antigen-induced murine B cell lymphomas. II. Exploitation of the surface idiotype as tumour specific antigen. *J. Immunol.*, **121**, 2358.

HOUGH, D. W., EADY, R. P., HAMBLIN, T. J., STEVENSON, F. K. & STEVENSON, G. T. (1976) Anti-idiotype raised against surface immunoglobulin of human neoplastic lymphocytes. *J. Exp. Med.*, **144**, 960.

HUGLI, T. E. & MÜLLER-EBERHARD, H. J. (1978) Anaphylatoxins: C3a and C5a. *Adv. Immunol.*, **26**, 1.

JOHANSSON, B., KLEIN, E. & HAGLUND, S. (1976) Correlation between the presence of surface localised immunoglobulin (Ig) and the histological type of human malignant lymphomas. *Clin. Immunol. Immunopathol.*, **5**, 119.

KASSEL, R. L., OLD, L. J., CARSWELL, E., FIORE, N & HARDY, W. D. (1973) Serum-mediated leukaemia cell destruction in AKR mice. *J. Exp. Med.*, **138**, 925.

KROLICK, K. A., ISAKSON, P. C., UHR, J. W. & VITETTA, E. S. (1979) $BCL_1$, a murine model for chronic lymphocytic leukaemia: Use of the surface immunoglobulin idiotype for the detection and treatment of tumour. *Immunol. Rev.*, **48**, 81.

LEECH, J. H., GLICK, A. D., WALDRON, J. A., FLEXNER, J. M., HORN, R. G. & COLLINS, R. D. (1975) Malignant lymphomas of follicular centre cell origin in man. I. Immunoglobulin studies. *J. Natl Cancer Inst.*, **54**, 11.

MACLENNAN, I. C. M. (1972) Antibody in the induction and inhibition of lymphocyte cytotoxicity. *Transplant Rev.*, **13**, 67.

MANASTER, J., FRÜHLING, J. & STRYCKMANS, P. (1973) Kinetics of lymphocytes in chronic lymphocytic leukaemia. I. Equilibrium between blood and a "readily accessible pool". *Blood*, **41**, 425.

PORATH, J., AXEN, R. & ERNBACK, S. (1967) Chemical coupling of proteins to agarose. *Nature*, **215**, 1491.

PROCTOR, J. W., RUDENSTAM, C. M. & ALEXANDER, P. A. (1973) A factor preventing the development of lung metastases in rats with sarcomas. *Nature*, **242**, 29.

SCOTT, J. L., MCMILLAN, R., MARINO, J. V. & DAVIDSON, J. G. (1973) Leukocyte labeling with $^{51}$chromium. IV. The kinetics of chronic lymphocytic leukemic lymphocytes. *Blood*, **41**, 155.

STEVENSON, F. K. & ELLIOTT, E. V. (1978) Mediation of cytotoxic functions by classes and subclasses of sheep antibody reactive with cell surface immunoglobulin idiotypic and constant region determinants. *Immunology*, **34**, 353.

STEVENSON, F. K., ELLIOTT, E. V. & STEVENSON, G. T. (1977*a*) Some effects on leukaemic B lymphocytes of antibodies to defined regions of their surface immunoglobulins. *Immunology*, **32**, 549.

STEVENSON, G. T., ELLIOTT, E. V. & STEVENSON, F. K. (1977*b*) Idiotypic determinants on the surface immunoglobulin of neoplastic lymphocytes: A therapeutic target. *Fed. Proc.*, **36**, 2268.

STEVENSON, G. T. & STEVENSON, F. K. (1975) Antibody to a molecularly-defined antigen confined to a tumour cell surface. *Nature*, **254**, 714.

STRONG, R. & WATKINS, J. (1979) A simple electrophoretic technique for the estimation of complement C3 conversion: Specific application to the investigation of anaphylactoid response to i.v. agents. *J. Immunol. Methods*, **29**, 293.

THEML, H., TREPEL, F., SCHICK, P., KABOTH, W. & BEGEMANN, H. (1973) Kinetics of lymphocytes in chronic lymphocytic leukemia: Studies using continuous $^{3}$H-thymidine infusion in two patients. *Blood*, **42**, 623.

VIRJI, M. & STEVENSON, G. T. (1979) Antibody-induced changes in levels of cyclic adenosine monophosphate in leukaemic lymphocytes. *Br. J. Cancer*, **39**, 434.